# Association of prophylactic transcatheter arterial embolization with perioperative blood loss and operative time in complex acetabular fractures: a propensity score-matched retrospective study

**DOI:** 10.3389/fmed.2026.1866547

**Published:** 2026-08-05

**Authors:** Bin Chen, Ningning Shen, Xinbo Gu, Jiapeng Wang, Ze Zhang, Mingming Kang, Peilu Shi, Yonghong Zhang, Huan Wang

**Affiliations:** 1 Second Clinical Medical College, Shanxi Medical University, Taiyuan, China; 2 Department of Orthopedics, Second Hospital of Shanxi Medical University, Taiyuan, China

**Keywords:** acetabular fracture, occult vascular injury, perioperative blood loss, propensity score matching, prophylactic transcatheter arterial embolization, superselective embolization

## Abstract

**Objective:**

To evaluate the association of preoperative prophylactic transcatheter arterial embolization (PTAE) with perioperative blood loss and operative time in hemodynamically stable acetabular fractures, and to explore outcomes associated with different embolization strategies.

**Methods:**

We retrospectively analyzed 304 patients treated between January 2023 and March 2025, including 106 who underwent PTAE and 198 who underwent open reduction and internal fixation without preoperative embolization. Propensity score matching generated 89 matched pairs. Intraoperative blood loss, total drainage output, hidden blood loss, total blood loss, and operative time were compared. Separate multivariable linear regression models were used for total blood loss and operative time. Within the PTAE cohort, outcomes were also compared between superselective superior gluteal artery (SGA) embolization and internal iliac artery (IIA) trunk embolization.

**Results:**

Angiography identified occult vascular injuries in 15 of 106 PTAE patients (14.15%). After matching, PTAE was associated with lower intraoperative blood loss, total drainage output, hidden blood loss, and total blood loss, as well as shorter operative time (all *p* < 0.001). Multivariable analyses confirmed independent associations with lower total blood loss (*β* = −204.71, *p* < 0.001) and shorter operative time (*β* = −22.116, *p* < 0.001). In exploratory subgroup analysis, blood loss outcomes and operative time did not differ significantly between the SGA and IIA groups, whereas the SGA group had lower 24-h postoperative VAS scores (4.19 ± 1.90 vs. 6.03 ± 2.01, *p* < 0.001).

**Conclusion:**

In this propensity score-matched retrospective cohort, preoperative PTAE was associated with lower perioperative blood loss and shorter operative time. Superselective embolization of anatomically high-risk vessels may be a potential strategy for perioperative vascular management, but subgroup findings should be interpreted cautiously.

## 1 Introduction

Pelvic and acetabular fractures (PAFs) account for approximately 1.5–3% of all fractures (1) and typically result from high-energy trauma. Due to their complex anatomy and proximity to major vascular and visceral structures, PAFs often occur in the setting of polytrauma and are associated with substantial morbidity and mortality, particularly in the setting of hemorrhagic shock (8–37%) (2, 3), with acute major hemorrhage representing a leading cause of early mortality (4). In hemodynamically unstable patients, transcatheter arterial embolization (TAE) has become an important emergency hemostatic intervention owing to its precision and efficacy (5, 6).

However, clinically assessed hemodynamic stability may not always reflect the absence of vascular injury, as a subset of patients may harbor occult vascular injuries—including arterial contusion, laceration, or pseudoaneurysm formation—that may become clinically significant during subsequent elective surgery (7, 8). When performing elective open reduction and internal fixation (ORIF), surgical maneuvers may disrupt the temporary tamponade effect provided by the hematoma, cause secondary vascular injury, or precipitate pseudoaneurysm rupture (9). These mechanisms may result in unexpected intraoperative hemorrhage and increase the complexity of surgical management.

Furthermore, while the role of TAE in emergency settings is widely acknowledged (10), existing guidelines and clinical practice primarily focus on its application in hemodynamically unstable patients, with comparatively less attention paid to perioperative bleeding prophylaxis in hemodynamically stable patients. This gap raises two important clinical considerations. Firstly, multidisciplinary management strategies (11) may prioritize acute stabilization, whereas the optimal conditions for subsequent definitive orthopedic fixation require separate consideration. For instance, emergency interventions for associated injuries may influence subsequent surgical access planning. Secondly, embolization is traditionally performed mainly for confirmed active bleeding (12), while the potential role of prophylactic embolization for securing high-risk vessels identified according to fracture anatomy and planned surgical approach remains insufficiently explored. This reactive approach may increase intraoperative uncertainty and perioperative management complexity.

Consequently, we investigated the clinical value of advancing the timing of interventional embolization, shifting the management approach from reactive treatment toward proactive preoperative risk control in high-risk but hemodynamically stable patients. We conducted a retrospective, propensity score-matched (PSM) study to evaluate the associations of preoperative prophylactic transcatheter arterial embolization with perioperative blood loss and surgical outcomes. In addition, we compared different embolization strategies to assess their associations with postoperative pain and other clinical outcomes. This study aimed to provide clinical insights into balancing hemostatic efficacy and minimization of non-target tissue injury in the management of complex acetabular fractures.

## 2 Materials and methods

### 2.1 Study design and participants

This single-center retrospective cohort study was approved by the Medical Ethics Committee of the Second Hospital of Shanxi Medical University (Approval No. [2025]YX-437) and was conducted in accordance with the Declaration of Helsinki. Written informed consent for clinical procedures, including PTAE and subsequent ORIF, was obtained from all patients before treatment. A total of 517 consecutive patients with pelvic or acetabular fractures admitted between January 2023 and March 2025 were initially screened. Based on the inclusion and exclusion criteria (Section 2.2), 213 patients were excluded due to isolated pelvic ring injuries, severe polytrauma requiring emergency intervention, or incomplete clinical records. In this study, hemodynamic stability was defined as the absence of ongoing hemorrhagic shock requiring emergency resuscitation or urgent hemostatic intervention before definitive fixation, allowing patients to proceed with planned elective surgical management. This definition was adopted to reduce the potential confounding effects of acute physiological instability on perioperative outcomes. Consequently, 304 eligible patients with acetabular fractures were enrolled. Among them, 106 patients received prophylactic transcatheter arterial embolization (PTAE) 1–3 days before planned ORIF (PTAE group), with target vessels selected according to fracture morphology, planned surgical exposure, and angiographic findings, while the remaining 198 patients underwent ORIF directly without preoperative embolization (Non-PTAE group). The decision to perform PTAE was not randomized and was not based on a prospectively validated standardized selection algorithm. Instead, treatment allocation followed a clinical and anatomy-guided selection framework incorporating fracture morphology, the anatomical region involved, the anticipated surgical approach and exposure, and multidisciplinary clinical judgment regarding perioperative bleeding risk. To minimize selection bias and ensure baseline comparability, propensity score matching (PSM) (13) was performed using a 1:1 nearest-neighbor matching algorithm with a caliper width of 0.1 standard deviations of the logit of the propensity score. Matching covariates included age, sex, body mass index (BMI), hypertension, diabetes, smoking history, anticoagulant use, Injury Severity Score (ISS), Letournel–Judet classification, concomitant pelvic ring injury (Tile classification), and time from injury to ORIF (days). The resulting matched pairs were used for the primary comparative analysis between the PTAE and Non-PTAE groups. Surgical approaches were selected based on fracture morphology and preoperative three-dimensional CT imaging, whereas target vessels for embolization were selected according to fracture morphology, planned surgical exposure, angiographic findings, and multidisciplinary discussion between the orthopedic and interventional radiology teams.

### 2.2 Inclusion and exclusion criteria

#### 2.2.1 Inclusion criteria

Patients were included if they met all the following criteria: (1) Age ≥18 years; (2) Radiographically confirmed traumatic acetabular fracture, classified according to the Letournel–Judet system by preoperative computed tomography (CT) and X-ray; (3) hemodynamic stability sufficient to undergo planned elective ORIF; (4) Eligible for ORIF with no absolute surgical contraindications.

#### 2.2.2 Exclusion criteria

Patients were excluded if they met any of the following criteria: (1) Requirement for emergency resuscitation or urgent hemostatic intervention before definitive fixation (e.g., hemorrhagic shock requiring resuscitation or emergency vascular embolization for pelvic/acetabular fracture-related bleeding); (2) Open pelvic or acetabular fractures; (3) Morel–Lavallée lesion involving the hip or pelvic region; (4) Known coagulopathy, pre-existing bleeding disorders, or systemic hematological diseases; (5) Documented preoperative allergy to contrast media or significant renal impairment (creatinine clearance <30 mL/min); (6) Neglected traumatic acetabular fractures (time from injury to admission >3 weeks); (7) Pathological acetabular fractures (e.g., caused by tumor, infection, or metabolic bone disease); (8) Active local or systemic infection; (9) Concomitant life-threatening injuries requiring prioritized emergency intervention (e.g., severe traumatic brain injury, major thoracic/abdominal/spinal organ damage); (10) Incomplete clinical records or insufficient radiological data.

**Figure 1:**
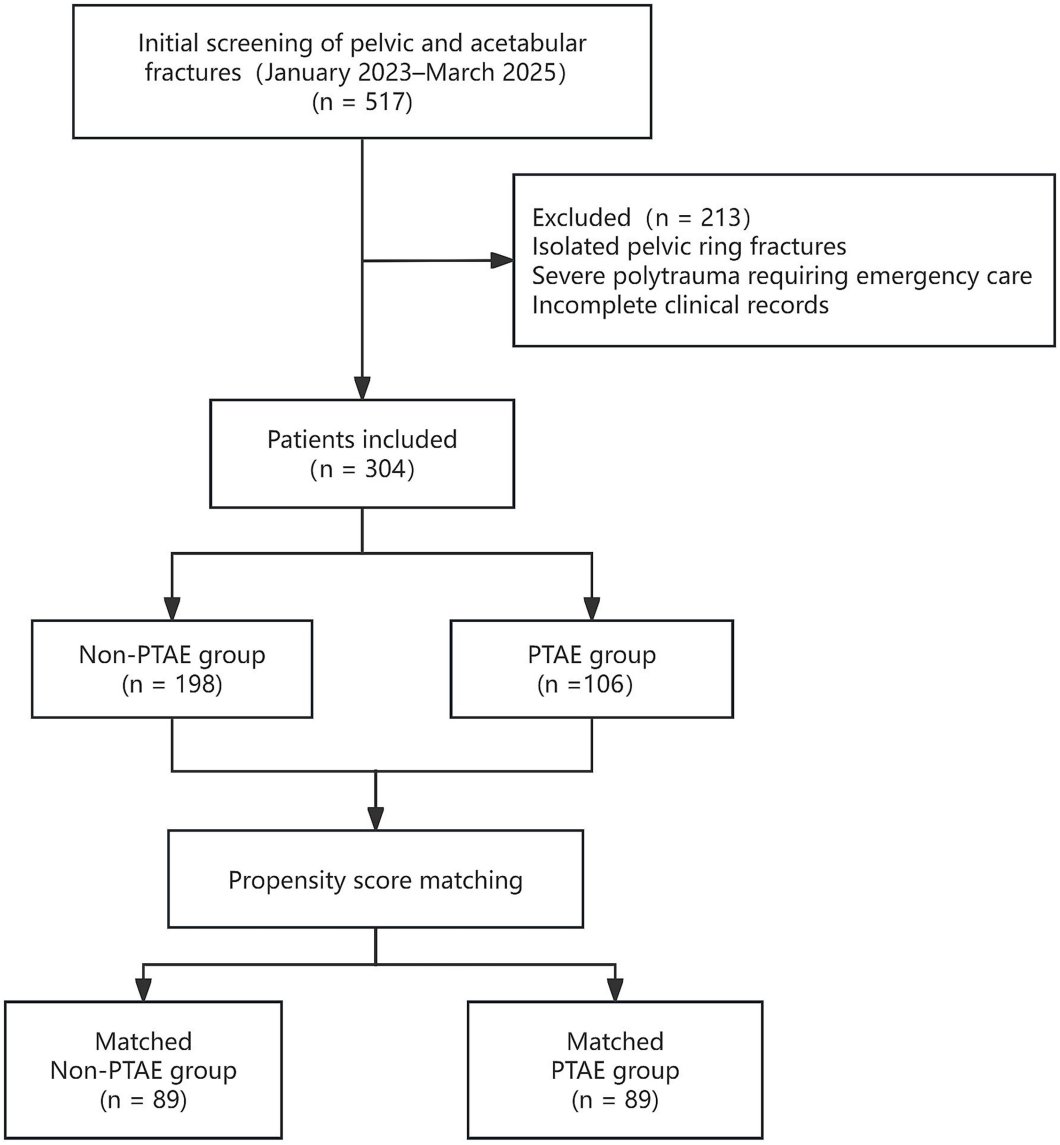
Flowchart of patient selection and propensity score matching.

**Figure 2:**
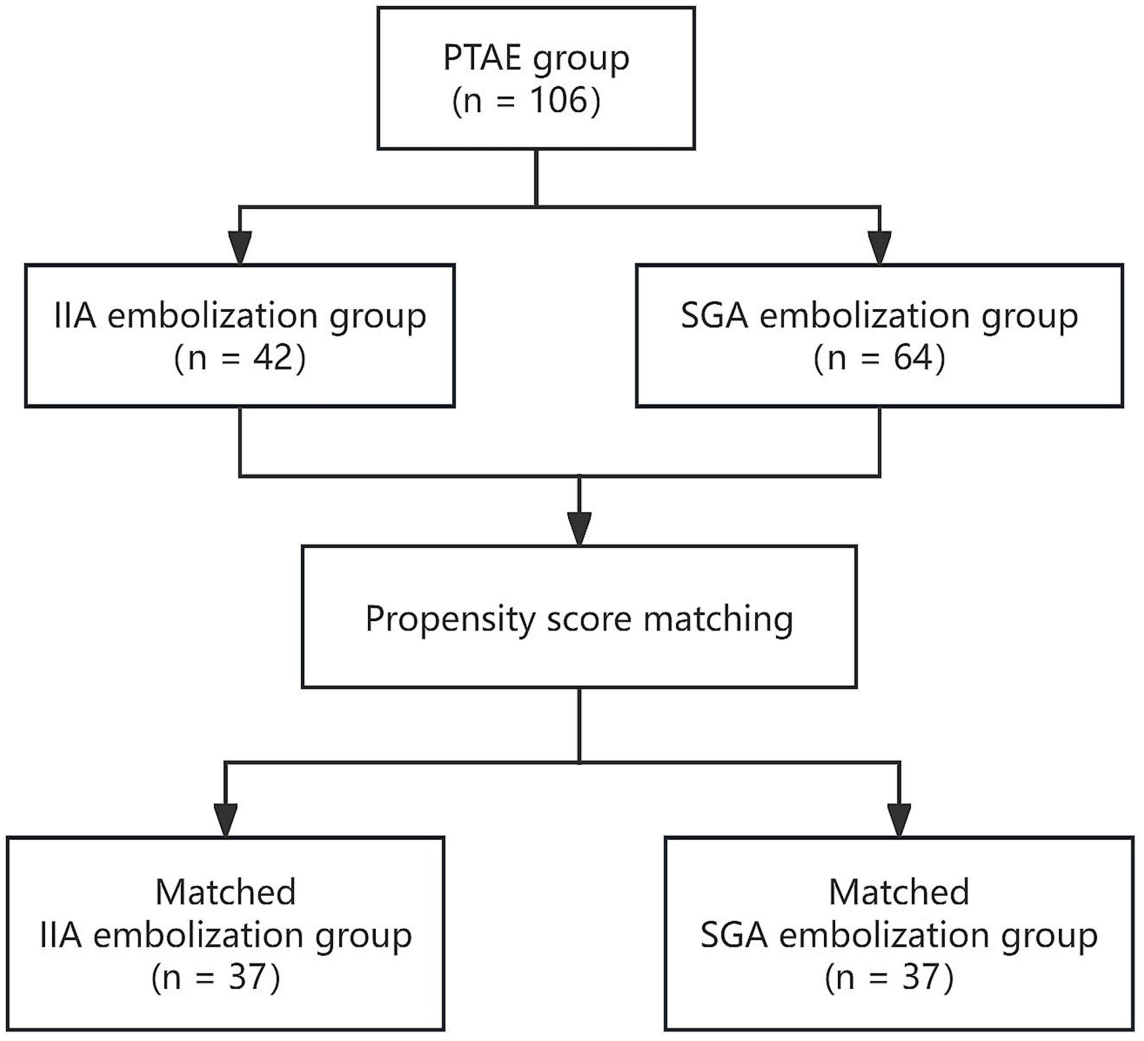
Flowchart of propensity score matching in the PTAE subgroup.

The patient selection process is shown in Figure 1, and the propensity score matching process is presented in Figure 2.

### 2.3 Assessment of hemodynamic stability

In this study, hemodynamic stability was defined pragmatically as the ability to proceed with planned elective fixation without requiring emergency resuscitation or urgent hemostatic intervention. The following physiological, perfusion, and laboratory parameters were considered collectively to evaluate clinical stability. These criteria were used to identify a relatively homogeneous elective surgical population and were not intended to represent absolute thresholds applicable to all trauma settings.

#### 2.3.1 Physiological and hemodynamic parameters

(1) Systolic blood pressure (SBP) ≥ 90 mmHg.
(2) Heart rate (HR) < 100 bpm.
(3) Shock index (SI; calculated as HR/SBP) < 0.8.
(4) Pulse pressure >30 mmHg.
(5) Respiratory rate (RR) between 12 and 20 breaths per minute.
(6) Core body temperature >35 °C.

#### 2.3.2 Tissue perfusion indices

(1) Urine output >0.5 mL/kg/h.
(2) Capillary refill time (CRT) < 2 s.

#### 2.3.3 Laboratory indicators

(1) Stable serial complete blood counts (CBC), interpreted together with clinical findings, without evidence of ongoing clinically significant hemorrhage based on hemoglobin (Hb) and hematocrit (Hct) trends.
(2) Arterial blood gas (ABG) findings without evidence of significant metabolic disturbance, generally reflected by serum lactate <2.0 mmol/L and base excess (BE) >−5 mmol/L.
(3) No clinically significant coagulation abnormalities requiring correction before elective fixation, with PT, aPTT, fibrinogen, and platelet count evaluated according to institutional reference ranges.

### 2.4 Fracture classification, surgical planning, and interventional protocol

#### 2.4.1 Fracture classification and surgical planning

**Figure 3:**
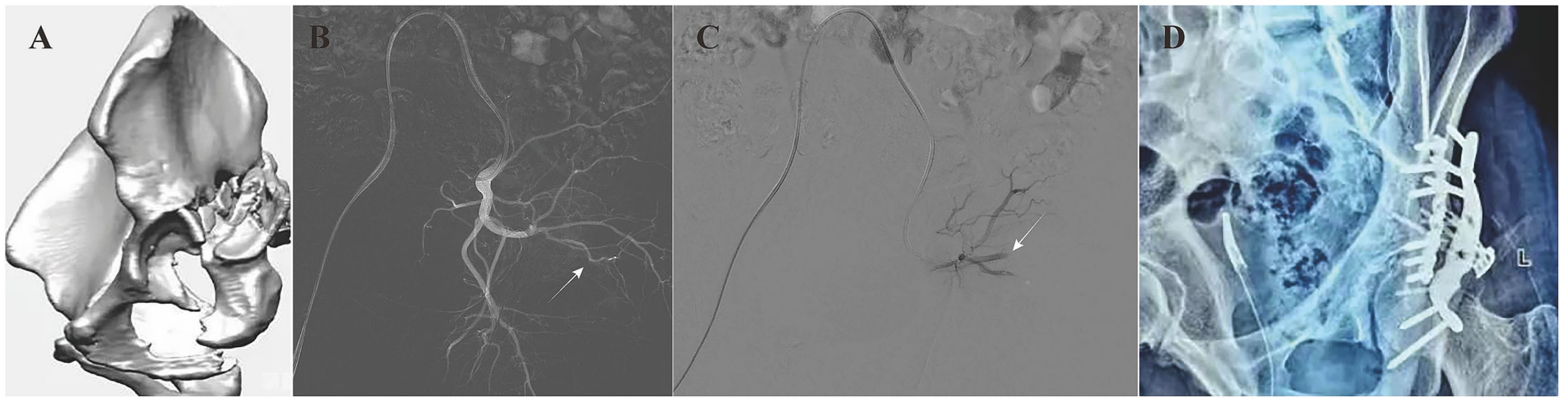
Representative case demonstrating anatomy-guided prophylactic superselective embolization in a complex acetabular fracture. **(A)** Three-dimensional CT reconstruction showing the acetabular fracture morphology and its anatomical relationship with the planned surgical approach. **(B)** Preoperative angiography demonstrating occult injury of a superior gluteal artery branch (white arrow). **(C)** Superselective catheterization and embolization of the injured superior gluteal artery branch, with the white arrow indicating the target vessel. **(D)** Postoperative radiograph following open reduction and internal fixation.

Preoperative imaging studies, including digital radiographs and three-dimensional CT reconstructions, were independently evaluated by two senior orthopedic surgeons. In cases of discrepancy, a third senior surgeon was consulted for a final assessment. Concomitant pelvic ring injuries were classified according to the Tile classification (types B and C were included), while acetabular fractures were categorized using the Letournel–Judet classification (including both elementary and associated types). The definitive surgical approach (e.g., Kocher–Langenbeck approach (14), ilioinguinal approach (15), modified Stoppa approach (16), or pararectus approach (17)) was determined collectively by the orthopedic team based on the three-dimensional fracture morphology and reduction strategy. All open reduction and internal fixation (ORIF) procedures were performed by senior orthopedic surgeons to reduce operator-related variability. The relationship between fracture patterns, surgical approaches, potential high-risk vessels, and target vessels considered for embolization is summarized in Supplementary Table S1. Representative correlations among fracture morphology, angiographic findings, and embolization procedures are shown in Figure 3.

#### 2.4.2 Prophylactic transcatheter arterial embolization technique

Prophylactic transcatheter arterial embolization (PTAE) was performed 1–3 days before the scheduled ORIF. This interval between embolization and definitive fixation was selected based on both biological and clinical considerations, allowing sufficient stabilization of the embolized vascular bed and establishment of reliable hemostatic control before surgical manipulation, while avoiding unnecessary delay of fracture fixation.

Target Selection: Target vessels were selected according to fracture morphology, the anatomical region involved, the planned surgical approach and exposure, and angiographic findings. The aim was to reduce the anticipated risk of major intraoperative bleeding from vessels located adjacent to the fracture or surgical field. Preoperative transcatheter arterial embolization was performed using either selective/superselective branch embolization or internal iliac artery trunk embolization according to fracture-related vascular risk and angiographic findings. For branch-level embolization, a microcatheter was advanced selectively or superselectively into the intended target vessel according to vessel anatomy and embolization strategy. Potential targets included the IIA trunk; branches of the IIA, such as the superior gluteal artery (SGA) and obturator artery; and relevant arterial anastomotic variants, including the corona mortis.

Embolization Strategy and Materials: The embolic agent was selected according to the target vessel diameter, angiographic findings, and the intended embolization endpoint.

(1) Gelatin sponge particles (350–560 μm): The particles were mixed with iodinated contrast medium to form a suspension and slowly injected through the microcatheter under continuous fluoroscopic monitoring until the predetermined angiographic endpoint, generally near-stasis of flow, was achieved.
(2) Metallic coils: Coils were used when permanent occlusion or adjunctive reinforcement was considered necessary, including cases of vessel transection, pseudoaneurysm formation, or inadequate occlusion with gelatin sponge particles alone.

Angiographic Assessment Criteria: During prophylactic angiography, the angiograms were assessed for the following abnormal findings: (1) active contrast extravasation, indicating active hemorrhage; (2) pseudoaneurysm formation, abrupt vessel cut-off, or intraluminal filling defects; (3) traumatic arteriovenous fistula; and (4) focal vascular engorgement, abnormal dilation, or abnormal plexiform branching adjacent to the fracture line, which were considered suspicious findings of occult vascular injury.

### 2.5 Assessment of perioperative blood loss

To comprehensively evaluate perioperative blood loss associated with prophylactic embolization in complex acetabular fractures, this study employed a standardized methodology to quantify measured blood loss (MBL), hidden blood loss (HBL), and total blood loss (TBL).

#### 2.5.1 Measured blood loss (MBL)

Measured blood loss (MBL) was defined as the directly measurable blood volume lost during and after surgery and was calculated as the sum of intraoperative blood loss (IBL) and total drainage output (TDO) using the following formula:


$$\begin{array}{c} \mathrm{MBL}=\text{Intraoperative Blood Loss}\;(\mathrm{IBL})\\ +\text{Total Drainage Output}\;(\mathrm{TDO}) \end{array}$$

(1) Intraoperative Blood Loss (IBL): IBL was calculated as the total volume of fluid collected in suction containers minus the total volume of irrigation fluid used, plus the estimated blood content in surgical gauze. To maintain practicality and consistency, the blood volume in gauze was estimated using a standardized visual estimation protocol (18) performed by a single senior circulating nurse to minimize inter-observer variability. The specific assessment criteria were as follows: Fully saturated gauze (4-ply): estimated as 20 mL; moderately soaked gauze (>50% area): estimated proportionally as 10 mL; minimally stained gauze (<50% area): estimated as 5 mL. These estimates were recorded in real time on both the surgical and anesthesia records.
(2) Total Drainage Output (TDO): TDO was defined as the total volume of fluid collected in the drainage system from the end of the surgery until drain removal.

#### 2.5.2 Total blood loss (TBL) and hidden blood loss (HBL)

Given the complex anatomy of the pelvic region and the propensity for postoperative blood accumulation in tissue compartments, the Gross hemoglobin balance method (19) was used to estimate total blood loss (TBL) and subsequently derive hidden blood loss (HBL). The calculations proceeded as follows:

Predicted Blood Volume (PBV): PBV was calculated using the Nadler formula:


$$\text{For males}:\mathrm{PBV}\;(\mathrm{mL})=(\begin{array}{l} 0.3669\times H^{3}\\ +0.03219\times W+0.6041 \end{array})\times 1,000$$

$$\text{For females}:\mathrm{PBV}\;(\mathrm{mL})=(\begin{array}{l} 0.3561\times H^{3}+0.03308\\ \times W+0.1833 \end{array})\times 1,000$$

Where *H* is height in meters, and W is weight in kilograms.

Calculated Total Blood Loss (TBL): TBL was calculated based on the change in hematocrit (Hct):


$$\mathrm{TBL}=\mathrm{PBV}\times \frac{{\mathrm{Hct}}_{\mathrm{pre}}-{\mathrm{Hct}}_{\text{post}}}{{\mathrm{Hct}}_{\mathrm{avg}}}$$

- Hct_pre: preoperative hematocrit.
- Hct_post: hematocrit measured on the morning of postoperative day 3.
- Hct_avg: the average of the preoperative and postoperative hematocrit values.

Derived Hidden Blood Loss (HBL): HBL was then derived using the formula:


$$\begin{array}{c} \mathrm{HBL}\;(\mathrm{mL})=\mathrm{TBL}-\mathrm{MBL}+\text{Volume of Allogeneic}\;\mathrm{Red}\;\\ \text{Blood Cell Transfusion} \end{array}$$

For calculation purposes, one unit of allogeneic packed red blood cells was standardized as 200 mL in this study.

### 2.6 Complication monitoring and subgroup pain assessment

#### 2.6.1 Complication monitoring

Intervention-specific complications (20) were recorded, including puncture site hematoma, post-procedural pseudoaneurysm, symptomatic gluteal muscle ischemia, local infection, and pelvic organ ischemia/necrosis.

#### 2.6.2 Pain assessment for the IIA trunk vs. SGA subgroups

Pain intensity was quantified using the Visual Analog Scale (VAS) (21), where a score of 0 represents “no pain” and 10 represents “unbearable pain.” Assessments were performed by a single senior nurse who was blinded to patient group allocation.

Assessment Time Point: The resting VAS score was recorded at 24 h postoperatively.

Subgroup Comparison: Within the PTAE group, postoperative pain levels were compared between patients who underwent superselective embolization of SGA branches and those who underwent IIA trunk embolization.

### 2.7 Statistical analysis

All statistical analyses were performed using SPSS software (version 25.0). Missing data were assessed before statistical analysis. Baseline characteristics, including age, sex, body mass index (BMI), comorbidities, and smoking history, were complete for all included patients. For variables with missing values (<10%), including continuous laboratory and clinical outcome variables, mean imputation was applied to preserve the complete sample size for subsequent analyses. No patients were excluded because of missing data, and complete-case analysis was not performed. The normality of continuous variables was assessed using the Shapiro–Wilk test. Normally distributed variables were presented as mean ± standard deviation (SD) and compared between groups using the independent samples t-test. Non-normally distributed variables were expressed as median (interquartile range) and compared using the Mann–Whitney U test. Categorical variables were described as frequency (percentage) and compared using the Pearson chi-square test or Fisher’s exact test, as appropriate. To control for potential selection bias inherent in the retrospective design, propensity score matching (PSM) was employed to balance baseline characteristics between the PTAE and Non-PTAE groups. Matching variables included age, sex, body mass index (BMI), hypertension, diabetes, smoking history, anticoagulant use, Injury Severity Score (ISS), Letournel–Judet classification, concomitant pelvic ring injury, and time from injury to surgery. A 1:1 nearest-neighbor matching algorithm with a caliper width of 0.1 standard deviations of the logit of the propensity score was applied. The matched cohort was used for the primary outcome analysis.

To identify independent predictors of total blood loss (TBL) and operative time (OT), separate univariable and multivariable linear regression analyses were performed for each outcome. Variables including age, sex, body mass index (BMI), hypertension, diabetes, smoking history, ISS, Letournel–Judet classification, history of anticoagulant use, and PTAE status were initially evaluated in univariable analyses. Prespecified clinically relevant variables were entered into the corresponding multivariable linear regression models. Because TBL and OT represent related perioperative outcomes and may introduce collinearity when included in the same regression model, they were analyzed separately. Furthermore, subgroup analyses were performed within the PTAE cohort. Because embolization strategy selection was not randomized, internal subgroup PSM was applied to reduce baseline imbalance between patients undergoing superselective superior gluteal artery (SGA) embolization and those undergoing internal iliac artery (IIA) trunk embolization. A 1:1 nearest-neighbor matching algorithm with a caliper width of 0.1 standard deviations of the logit of the propensity score was used. After matching, differences in blood loss outcomes and postoperative Visual Analog Scale (VAS) pain scores were compared between these subgroups. For all statistical analyses, a two-sided *p* < 0.05 was considered statistically significant.

## 3 Results

### 3.1 Angiographic findings during prophylactic embolization

Among the 106 patients who underwent preoperative prophylactic transcatheter arterial embolization (PTAE) with DSA guidance, 15 (14.15%) had occult traumatic vascular injuries, involving a total of 17 vascular lesions. These vascular injuries manifested as contrast extravasation or pseudoaneurysm formation. Single-vessel injury was the predominant pattern (13/15, 86.7%), with the superior gluteal artery (SGA) being the most frequently involved vessel (8/15, 53.3%). Multiple-vessel involvement was identified in 2 patients (13.3%). Detailed distributions of the injured vessels and vascular injury patterns are presented in Supplementary Table S2, and representative angiographic findings of different occult vascular injuries are shown in Supplementary Figure S1.

### 3.2 Study cohort and propensity score matching results

**Figure 4:**
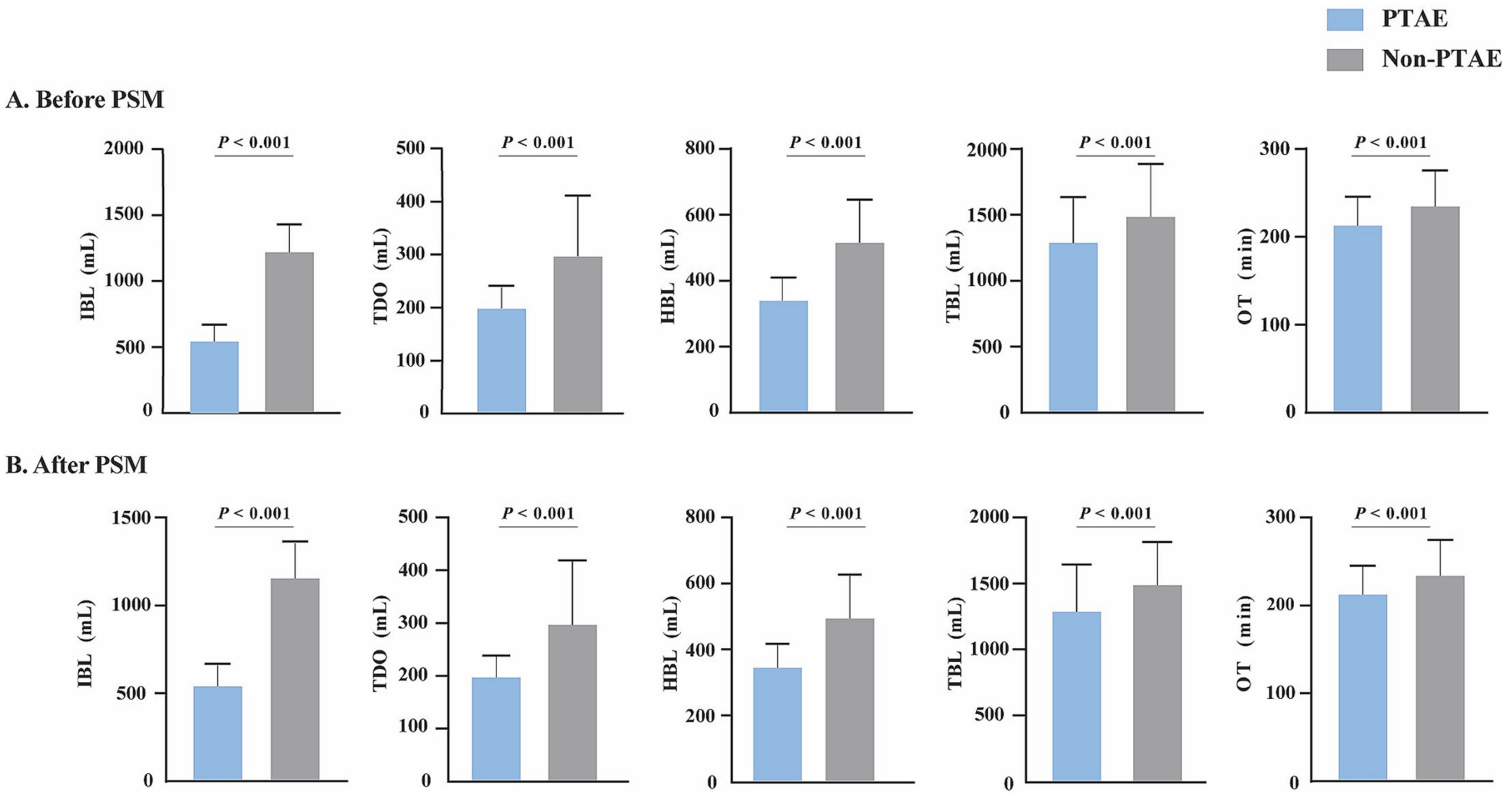
Comparison of perioperative outcomes between the PTAE and Non-PTAE groups before and after propensity score matching (PSM). Data are presented as mean ± standard deviation.

This study initially included 304 patients with acetabular fractures, of whom 106 underwent preoperative prophylactic transcatheter arterial embolization (PTAE group) and 198 underwent ORIF without preoperative embolization (Non-PTAE group). Before matching, significant differences were observed between the two groups in BMI, hypertension, smoking history, and Injury Severity Score (ISS) (all *p* < 0.05). To reduce potential selection bias, propensity score matching (PSM) was performed based on variables including age, sex, body mass index (BMI), hypertension, diabetes, smoking history, anticoagulant use, Injury Severity Score (ISS), concomitant pelvic ring injury, Letournel–Judet fracture classification, and time from injury to ORIF (days). Using a 1:1 nearest-neighbor matching algorithm with a caliper width of 0.1 standard deviations of the logit of the propensity score, 89 patients from the PTAE group were successfully matched with 89 patients from the Non-PTAE group, resulting in a balanced cohort of 178 patients for subsequent analyses. After matching, no statistically significant differences remained between the two groups in baseline characteristics (all *p* > 0.05). Detailed comparisons of baseline characteristics before and after PSM are presented in Table 1, while comparisons of perioperative outcomes are shown in Figure 4.

**Table 1:** Baseline characteristics and perioperative outcomes of patients in the non-PTAE and PTAE groups before and after propensity score matching.

Characteristic	Before PSM			After PSM		
	Non-PTAE group (*n* = 198)	PTAE group (*n* = 106)	*p* value	Non-PTAE group (*n* = 89)	PTAE group (*n* = 89)	*p* value
Age (y)	46 (43–50)	47 (44–51)	0.438	46 (42–50)	47 (44–50)	0.213
BMI	26.2 (25.2–27.1)	24.2 (21.7–26.7)	0.001	24.4 (21.4–27.0)	24.3 (20.9–27.3)	0.550
Sex			0.155			0.703
Male	136 (68.7)	81 (76.4)		71 (79.8)	73 (82.0)	
Female	62 (31.3)	25 (23.6)		18 (20.2)	16 (18.0)	
Hypertension	67 (33.8)	21 (19.8)	0.010	21 (23.6)	17 (19.1)	0.464
Diabetes	21 (10.6)	6 (5.7)	0.149	6 (6.7)	4 (4.5)	0.515
Smoke	77 (38.9)	29 (27.4)	0.044	21 (23.6)	25 (28.1)	0.493
Anticoagulant	28 (14.1)	11 (10.4)	0.350	7 (7.9)	9 (10.1)	0.600
ISS	16 (14–19)	17 (15–19)	0.002	16 (14–18)	17 (15–19)	0.059
Letournel–Judet classification			0.939			0.925
Posterior wall fracture	48 (24.2)	24 (22.6)		21 (23.6)	19 (21.3)	
Posterior column fracture	13 (6.6)	8 (7.5)		4 (4.5)	7 (7.9)	
Transverse fracture	17 (8.6)	11 (10.4)		7 (7.9)	10 (11.2)	
Posterior column with posterior wall fracture	12 (6.1)	6 (5.7)		5 (5.6)	6 (6.7)	
Transverse fracture with posterior wall fracture	26 (13.1)	17 (16.0)		17 (19.1)	14 (15.7)	
Anterior column with posterior hemitransverse fracture	11 (5.6)	4 (3.8)		6 (6.7)	4 (4.5)	
T-shaped fracture	19 (9.6)	13 (12.3)		9 (10.1)	11 (12.4)	
Both-column fracture	52 (26.3)	23 (21.7)		20 (22.5)	18 (20.2)	
Coexisting pelvic ring fractures	86 (43.4)	44 (41.5)	0.746	32 (36.0)	35 (39.3)	0.643
Time from injury to ORIF (days)	12.11 ± 2.71	11.46 ± 3.11	0.062	11.69 ± 2.59	11.29 ± 3.10	0.354
OT (min)	238.08 ± 39.85	214.84 ± 32.49	*p* < 0.001	235.99 ± 40.58	214.67 ± 32.63	*p* < 0.001
IBL (mL)	1,221.29 ± 203.59	558.03 ± 120.83	*p* < 0.001	1,165.04 ± 199.99	550.01 ± 119.99	*p <* 0.001
TDO (mL)	292.87 ± 114.22	201.59 ± 39.97	*p* < 0.001	300.01 ± 120.01	200.02 ± 39.97	*p* < 0.001
HBL (mL)	513.02 ± 127.03	345.18 ± 70.52	*p* < 0.001	499.98 ± 129.98	350.01 ± 69.96	*p* < 0.001
TBL (mL)	1,528.61 ± 411.12	1,300.02 ± 351.07	*p* < 0.001	1,500.02 ± 330.01	1,300.01 ± 359.98	*p* < 0.001

BMI, body mass index; ISS, injury severity score; ORIF, open reduction and internal fixation; OT, operative time; IBL, intraoperative blood loss; TDO, total drainage output; HBL, hidden blood loss; TBL, total blood loss; MBL, measured blood loss; PTAE, prophylactic transcatheter arterial embolization.

### 3.3 Comparison of perioperative outcomes

The perioperative data of the two groups after matching are detailed in Table 1. Regarding blood loss control, all metrics were significantly lower in the PTAE group compared to the Non-PTAE group. The PTAE group demonstrated significantly lower measured blood loss (MBL), including reduced intraoperative blood loss (IBL) (550.01 ± 119.99 mL vs. 1,165.04 ± 199.99 mL, *p* < 0.001) and total drainage output (TDO) (200.02 ± 39.97 mL vs. 300.01 ± 120.01 mL, *p* < 0.001). Furthermore, the PTAE group showed significantly lower hidden blood loss (HBL) (350.01 ± 69.96 mL vs. 499.98 ± 129.98 mL, *p* < 0.001), which was accompanied by significantly lower total blood loss (TBL) (1,300.01 ± 359.98 mL vs. 1,500.02 ± 330.01 mL, *p* < 0.001). In terms of surgical efficiency, the operative time was significantly shorter in the PTAE group (214.67 ± 32.63 min vs. 235.99 ± 40.58 min, *p* < 0.001). Safety evaluation revealed that no PTAE-related complications, including puncture site hematoma, post-procedural pseudoaneurysm, symptomatic gluteal muscle ischemia, local infection, or pelvic organ ischemia/necrosis, were observed in the PTAE group.

### 3.4 Multivariable regression analysis

To evaluate the independent association between prophylactic transcatheter arterial embolization (PTAE) and key perioperative outcomes, separate multivariable linear regression models were constructed for total blood loss (TBL) and operative time (OT) using both the pre- and post-propensity score matching (PSM) cohorts (Tables 2, 3). In the regression model for total blood loss, after adjustment for prespecified clinically relevant covariates, PTAE remained independently associated with lower TBL (post-PSM: *β* = −204.71, *p* < 0.001). A similar association was observed in the pre-PSM cohort (*β* = −194.529, *p* = 0.012). In the separate regression model for operative time, PTAE remained independently associated with shorter OT after adjustment for potential confounders (post-PSM: *β* = −22.116, *p* < 0.001). A similar association was observed in the pre-PSM cohort (*β* = −26.799, *p* < 0.001).

**Table 2:** Linear regression analysis of factors associated with total blood loss before and after propensity score matching.

Characteristic	Before PSM				After PSM			
	Univariable analysis		Multivariable analysis		Univariable analysis		Multivariable analysis	
	*β*	*p* value	*β*	*p* value	*β*	*p* value	*β*	*p* value
Sex	−15.021	0.756	2.970	0.952	−25.174	0.714	1.993	0.977
Age	2.849	0.407	4.834	0.153	−0.424	0.930	3.405	0.469
BMI	31.842	0.006	14.363	0.250	15.556	0.389	−5.001	0.789
Hypertension	−71.364	0.138	−14.003	0.897	−55.764	0.397	150.897	0.304
Diabetes	−77.904	0.328	61.575	0.650	−135.213	0.248	163.638	0.369
Smoke	−73.442	0.104	−39.383	0.683	−94.681	0.123	−128.601	0.322
History of anticoagulant use	−118.631	0.077	−107.747	0.391	−220.733	0.018	−326.324	0.044
ISS	6.575	0.265	10.321	0.079	8.842	0.256	15.381	0.079
Letournel–Judet classification	−9.562	0.198	−8.256	0.257	−9.989	0.332	−8.304	0.404
PTAE	−199.958	*P <* 0.001	−194.529	0.012	−200.011	*P <* 0.001	−204.711	*P <* 0.001

PSM, propensity score matching; PTAE, prophylactic transcatheter arterial embolization; *β*, regression coefficient.

**Table 3:** Linear regression analysis of factors associated with operative time (OT) before and after propensity score matching.

Variables	Before PSM				After PSM			
	Univariable analysis		Multivariable analysis		Univariable analysis		Multivariable analysis	
	*β*	*p* value	*β*	*p* value	*β*	*p* value	*β*	*p* value
Sex	−3.604	0.505	0.906	0.868	4.700	0.521	1.779	0.810
Age	−0.044	0.916	0.037	0.927	−0.351	0.541	−0.112	0.842
BMI	−0.176	0.783	−0.949	0.130	−0.757	0.264	−0.877	0.186
Hypertension	7.756	0.153	−11.008	0.365	15.739	0.024	3.764	0.812
Diabetes	−6.161	0.486	−22.966	0.141	13.635	0.096	−9.980	0.608
Smoke	9.921	0.052	23.641	0.030	12.188	0.875	8.335	0.555
History of anticoagulant use	−1.877	0.806	5.902	0.616	13.782	0.170	10.022	0.563
ISS	0.019	0.979	0.524	0.449	−0.748	0.442	−0.266	0.781
Letournel–Judet classification	0.466	0.577	0.473	0.556	−0.004	0.997	−0.113	0.916
PTAE	−23.236	*p <* 0.001	−26.799	*p <* 0.001	−21.315	*p <* 0.001	−22.116	*p <* 0.001

PSM, propensity score matching; PTAE, prophylactic transcatheter arterial embolization.

### 3.5 Subgroup comparison between superselective SGA and IIA trunk embolization

To explore the potential associations between embolization strategy and clinical outcomes, an exploratory subgroup analysis was conducted within the PTAE cohort comparing superselective superior gluteal artery (SGA) embolization (SGA group, *n* = 64) with internal iliac artery (IIA) trunk embolization (IIA group, *n* = 42). Internal subgroup propensity score matching was performed using the same propensity score model as the primary analysis. After matching, 37 matched pairs, comprising 74 patients in total, were obtained. Baseline characteristics before and after matching are detailed in Table 4. The subgroup analysis did not demonstrate statistically significant differences in perioperative blood loss outcomes or operative time between the two groups. After matching, intraoperative blood loss [547.81 (95% CI: 506.92–588.70) vs. 566.41 (95% CI: 526.54–606.27), *p* = 0.511], total drainage output [200.30 (95% CI: 185.68–214.92) vs. 204.27 (95% CI: 191.21–217.33), *p* = 0.682], hidden blood loss [347.97 (95% CI: 326.59–369.36) vs. 348.35 (95% CI: 323.54–373.16), *p* = 0.981], and total blood loss [1,296.03 (95% CI: 1,179.77–1,412.28) vs. 1,315.35 (95% CI: 1,191.24–1,439.46), *p* = 0.818] showed no statistically significant differences between the SGA and IIA groups. Similarly, no statistically significant difference was observed in operative time [211.9 (95% CI: 200.4–223.4) vs. 221.6 (95% CI: 211.8–231.4), *p* = 0.195]. The absence of statistically significant differences in blood loss outcomes or operative time should not be interpreted as evidence of equivalence between the two embolization strategies. However, a statistically significant difference was observed in postoperative pain outcomes. The SGA group demonstrated lower postoperative VAS pain scores compared with the IIA group (4.19 ± 1.90 vs. 6.03 ± 2.01, *p* < 0.001). These exploratory subgroup findings require further validation in larger prospective cohorts.

**Table 4:** Subgroup comparison between superselective superior gluteal artery and internal iliac artery trunk embolization before and after propensity score matching.

Characteristic	Before PSM			After PSM		
	IIA trunk embolization (*n* = 42)	SGA embolization (*n* = 64)	*p* value	IIA trunk embolization (*n* = 37)	SGA embolization (*n* = 37)	*p* value
Age (y)	48 (45–50)	47 (44–51)	0.439	48 (44–51)	46 (42–50)	0.395
BMI	25.2 (22.5–27.6)	23.9 (21.2–25.8)	0.099	25.8 (22.7–27.8)	23.8 (19.5–25.4)	0.029
Sex			0.609			0.425
Male	31 (73.8)	50 (78.1)		26 (70.3)	29 (78.4)	
Female	11 (26.2)	14 (21.9)		11 (29.7)	8 (21.6)	
Hypertension	8 (19.0)	13 (20.3)	0.873	8 (21.6)	4 (10.8)	0.207
Diabetes	1 (2.4)	5 (7.8)	0.237	1 (2.7)	1 (2.7)	0.999
Smoke	12 (28.6)	17 (26.6)	0.820	11 (29.7)	5 (13.5)	0.090
Anticoagulant	3 (7.1)	8 (12.5)	0.376	3 (8.1)	2 (5.4)	0.643
ISS	17.5 (15.0–20.0)	17 (15–19)	0.221	18 (15–20)	17 (15–19)	0.504
Letournel–Judet classification			0.542			0.273
Posterior wall fracture	7 (16.8)	17 (26.6)		6 (16.2)	12 (32.4)	
Posterior column fracture	4 (9.5)	4 (6.3)		4 (10.8)	1 (2.7)	
Transverse fracture	3 (7.1)	8 (12.5)		2 (5.4)	5 (13.5)	
Posterior column fracture with posterior wall fracture	1 (2.4)	5 (7.8)		1 (2.7)	3 (8.1)	
Transverse fracture with posterior wall fracture	8 (19.0)	9 (14.1)		7 (18.9)	5 (13.5)	
Anterior column fracture with posterior hemitransverse fracture	1 (2.4)	3 (4.7)		1 (2.7)	2 (5.4)	
T-shaped Fracture	6 (14.3)	7 (10.9)		5 (13.5)	3 (8.1)	
Both-column fracture	12 (28.6)	11 (17.2)		11 (29.7)	6 (16.2)	
Coexisting Pelvic ring fractures	17 (40.5)	27 (42.2)	0.861	15 (40.5)	13 (35.1)	0.632
Time from injury to ORIF (d)	10.90 ± 3.13	11.83 ± 3.07	0.133	10.82 ± 3.15	12.03 ± 3.09	0.100
Operative time (min)	221.19 ± 30.85	210.67 ± 33.11	0.103	221.59 ± 29.26	211.86 ± 34.53	0.195
Intraoperative blood loss (IBL)	573.95 ± 116.78	547.58 ± 123.19	0.274	566.41 ± 119.57	547.81 ± 122.63	0.511
Total drainage output (TDO)	206.71 ± 37.72	198.23 ± 41.31	0.287	204.27 ± 39.17	200.30 ± 43.85	0.682
Total blood loss (TBL)	1,296.43 ± 355.81	1,302.38 ± 350.72	0.933	1,315.35 ± 372.24	1,296.03 ± 348.68	0.818
Hidden blood loss (HBL)	351.19 ± 71.04	341.23 ± 70.45	0.480	348.35 ± 74.41	347.97 ± 64.14	0.981
VAS	5.98 ± 1.96	3.98 ± 2.05	*p <* 0.001	6.03 ± 2.01	4.19 ± 1.90	*p* < 0.001

## 4 Discussion

The key angiographic finding of this study is that 14.15% of patients, despite being strictly defined as hemodynamically stable, were found to have occult vascular injuries, including active hemorrhage or pseudoaneurysm formation. This finding challenges the assumption that hemodynamic stability alone indicates a low risk of occult vascular injury and supports a more proactive approach to vascular assessment before definitive fixation. Prophylactic transcatheter arterial embolization (PTAE) was associated with lower measured and hidden blood loss and shorter operative time. These associations may be related to improved surgical field visibility; however, causal effects cannot be established because of the retrospective, non-randomized study design.

Acetabular fractures present a formidable challenge due to their complex anatomy and proximity to major vascular and visceral structures. The clinical risk lies not only in the difficulty of reduction but also in the potential for unexpected major intraoperative hemorrhage (22). For instance, in posterior approaches, branches of the superior gluteal artery (SGA) (7, 23) course intimately along the bony surface and may retract into the greater sciatic notch upon injury, making traditional surgical ligation extremely difficult. Similarly, the corona mortis (24) encountered in anterior approaches poses an analogous hemorrhagic threat. Crucially, intraoperative bleeding may obscure the surgical field, prolong operative time, and increase the risk of postoperative surgical site infection (25). Therefore, controlling perioperative hemorrhage is paramount for improving perioperative safety. Our protocol—utilizing preoperative angiography to identify and treat occult vascular lesions alongside preemptive embolization of high-risk vessels—may reduce the risk of uncontrolled intraoperative bleeding and unexpected vascular complications. While preoperative balloon occlusion (26, 27) has been reported as an alternative strategy to mitigate bleeding, its clinical utility may be limited by a finite occlusion window and potential ischemic and vascular complications (28). In contrast, PTAE may provide a more controlled approach by securing target vessels before the first incision is made, thereby facilitating a safer and more predictable surgical environment. Recent studies of hip surgery for heterotopic ossification have likewise reported the use of preoperative arterial or combined arterial and venous embolization in patients at high risk of perioperative hemorrhage, further supporting the broader concept of preventive vascular control before surgery (29, 30).

Existing literature supports the use of prophylactic coil embolization (31), which has demonstrated effectiveness in reducing intraoperative blood loss and operative time in acetabular surgery. In our protocol, coils were reserved for cases requiring permanent occlusion or adjunctive reinforcement, including vessel transection, pseudoaneurysm formation, or inadequate occlusion with gelatin sponge particles alone. Gelatin sponge particles were used when temporary embolization was considered appropriate (32). The optimal timing between PTAE and definitive fixation remains an important consideration. In our protocol, embolization was performed 1–3 days before surgery rather than immediately before incision or several days in advance. This interval was selected based on both biological and clinical considerations. From a biological perspective, this interval allows sufficient stabilization of the embolized vascular bed and establishment of effective local hemostasis, while avoiding prolonged interruption of regional perfusion. From a clinical perspective, it provides a practical window for multidisciplinary coordination between interventional radiology and orthopedic teams, while avoiding unnecessary delay of definitive fracture fixation. A prolonged interval may theoretically increase the risk of recurrent bleeding due to collateral vessel recruitment or incomplete control of occult vascular lesions. In addition to timing optimization, embolic particle diameter is another critical factor influencing the balance between hemostatic efficacy and tissue preservation (33). In our protocol, larger-diameter particles (350–560 μm) were selected. These particles were intended to occlude the targeted high-risk branches while limiting distal microvascular penetration, thereby minimizing the risk of microvascular compromise in non-target tissues. This strategic design helps achieve a clear surgical field without permanently compromising regional blood supply, while preserving functional vascular integrity.

In the posterior Kocher–Langenbeck (K–L) approach, the superior gluteal artery (SGA) is the primary high-risk vessel for massive intraoperative hemorrhage. Previous anatomical studies have demonstrated that the SGA’s superficial branch primarily supplies the gluteus maximus, while its deep branch system—comprising the superior-deep, inferior-deep, supra-acetabular, and acetabular branches—courses between the gluteus medius and minimus to supply the posterior column, acetabular roof, and abductor musculature. During K–L exposure and reduction, the inferior-deep and supra-acetabular branches, which course intimately along the bony surface, are particularly vulnerable to injury. Our superselective strategy targets these core bleeding sources and may provide a potential balance between hemostatic efficacy and tissue preservation. By selectively avoiding unnecessary embolization of non-target branches, this approach may help preserve collateral circulation and surrounding muscle perfusion. This anatomy-guided intervention offers a possible mechanistic explanation for our clinical findings: the lower postoperative VAS scores observed in the SGA group compared with the IIA trunk group (4.19 ± 1.90 vs. 6.03 ± 2.01, *p* < 0.001) suggest that selective branch-level embolization may be associated with less postoperative discomfort than more extensive proximal embolization. Because pain was assessed only once at 24 h postoperatively, this finding should be regarded as exploratory and should not be extrapolated to sustained pain relief or long-term functional benefit. In addition, the pain analysis was not specifically adjusted for analgesic protocols, surgical approach, soft-tissue injury, fracture characteristics, or other perioperative pain-management factors; therefore, the observed VAS difference should not be interpreted as evidence of a causal analgesic effect of superselective embolization. The potential relationship between reduced non-target vascular compromise and postoperative pain requires confirmation in larger studies with standardized pain assessment and longer follow-up.

The clinical benefits of PTAE extend beyond controlling visible intraoperative hemorrhage. Given the deep anatomical location of the acetabulum and the large potential retroperitoneal space (34), perioperative hidden blood loss (HBL) (35) may be substantially underestimated in these patients. Our findings showed that PTAE was associated with lower intraoperative blood loss and HBL, potentially by limiting ongoing bleeding from injured vascular beds after surgery. Furthermore, multivariable regression analysis demonstrated that PTAE was independently associated with reduced total blood loss and shorter operative time. The improved operative conditions achieved through preoperative vascular intervention may facilitate fracture reduction and surgical exposure, thereby contributing to reduced operative duration. This potential interaction between bleeding control and operative efficiency may help optimize perioperative management and surgical workflow.

This study has several limitations that warrant consideration. First, this single-center retrospective study was inherently subject to selection bias and center-specific clinical practice patterns. PTAE allocation was not randomized and was based on a clinical and anatomy-guided selection framework rather than a prospectively validated standardized algorithm. Because fracture morphology, planned surgical approach and exposure, perceived bleeding risk, and multidisciplinary clinical judgment may have influenced both treatment allocation and perioperative outcomes, confounding by indication cannot be excluded. Although propensity score matching and multivariable regression analyses were used to reduce imbalance in measured covariates, these methods cannot completely eliminate selection bias or residual and unmeasured confounding. Therefore, the observed associations should be interpreted cautiously. Second, the subgroup analysis comparing SGA embolization with IIA trunk embolization included only 37 matched pairs and was exploratory. Therefore, the analysis may have been underpowered to detect clinically meaningful differences, and the absence of statistically significant findings should not be interpreted as evidence of equivalence between the two embolization strategies. In addition, postoperative pain was assessed using a single resting VAS score at 24 h, which precluded evaluation of temporal changes in pain, persistent pain, or longer-term functional outcomes. Potential confounding from analgesic protocols, surgical approach, soft-tissue injury, fracture characteristics, and perioperative pain management could not be fully accounted for because these variables were not comprehensively recorded or adjusted for in the subgroup analysis. Third, the follow-up duration was short and focused primarily on perioperative outcomes; therefore, long-term assessments of muscle strength recovery, chronic pain, functional outcomes, quality of life, and rare or delayed complications were lacking. Accordingly, the absence of recorded PTAE-related complications during the perioperative period should not be interpreted as evidence of long-term safety. Future large-scale multicenter prospective studies are needed to confirm the observed associations, further evaluate the safety and clinical value of this strategy, refine its clinical indications, and assess mid- to long-term functional outcomes.

In conclusion, in this propensity score-matched retrospective cohort, preoperative PTAE was associated with lower perioperative blood loss and shorter operative time in hemodynamically stable patients with acetabular fractures. Superselective embolization targeting anatomically high-risk vessels may represent a promising strategy for perioperative vascular management; however, the subgroup findings should be considered exploratory. Because residual and unmeasured confounding cannot be excluded, prospective multicenter studies with standardized treatment indications are required to confirm these findings.

## Data Availability

The raw data supporting the conclusions of this article will be made available by the authors, without undue reservation.
