# Supplementary material for: Association of prophylactic transcatheter arterial embolization with perioperative blood loss and operative time in complex acetabular fractures: a propensity score-matched retrospective study
Source: Front Med (Lausanne). 2026 Aug 5;13:1866547. doi: 10.3389/fmed.2026.1866547 (PMC13485805; doi:10.3389/fmed.2026.1866547)
Supplement: Supplementary file 1 [file Supplementary_file_1.DOCX]

### Supplementary Table S1.

**Relationship between acetabular fracture patterns, surgical approaches, potential high-risk vessels, and corresponding embolization targets identified according to fracture morphology, surgical approach, and angiographic findings.**

| **Fracture pattern** | **Typical surgical approach** | **Potential high-risk vessels** | **Corresponding target vessels for embolization** |
| --- | --- | --- | --- |
| Posterior wall and/or posterior column | Kocher–Langenbeck approach | Superior gluteal artery (SGA) branches | Deep branches of SGA, including supra-acetabular and acetabular branches |
| Anterior wall and/or anterior column | Ilioinguinal approach, lateral rectus approach (LRA), or pararectus approach (PRA) | Obturator artery branches, corona mortis vessels, and inferior epigastric artery branches | Corresponding injured or high-risk branches identified by angiography |
| Transverse | Approach selected according to fracture extension and surgical exposure requirements | SGA branches, obturator artery branches, and other approach-dependent branches of the internal iliac artery | Corresponding high-risk branches of the internal iliac artery identified by angiography |
| Both-column | Combined anterior and posterior approaches when required | Multiple branches of the internal iliac artery depending on anterior and posterior components | Individual high-risk branches of the internal iliac artery identified by angiography |

**Footnote:**
Target vessel selection was based on a comprehensive assessment of fracture morphology, planned surgical approach and exposure, and angiographic findings rather than fracture classification alone.

### Supplementary Table S2.

### ****Distribution of occult traumatic vascular injuries identified during preoperative prophylactic transcatheter arterial embolization.****

| **Vascular involvement pattern** | **Involved vessel(s)** | **Patients, n (%)** |
| --- | --- | --- |
| Single-vessel involvement |  | 13 (86.7%) |
|  | Superior gluteal artery (SGA) | 8 (53.3%) |
|  | Inferior gluteal artery (IGA) | 1 (6.7%) |
|  | Internal pudendal artery (IPA) | 2 (13.3%) |
|  | Obturator artery (OA) | 1 (6.7%) |
|  | Other pelvic arterial branch | 1 (6.7%) |
| Multiple-vessel involvement |  | 2 (13.3%) |
|  | Superior gluteal artery (SGA) and obturator artery (OA) | 1 (6.7%) |
|  | Internal pudendal artery (IPA) and obturator artery(OA) | 1 (6.7%) |

Note: Two patients had multiple vascular lesions; therefore, the total number of vascular lesions (17) exceeded the number of patients (15).

Supplementary Figure S1. Representative angiographic findings of occult vascular injuries identified during preoperative angiographic evaluation for prophylactic embolization in patients with acetabular fractures. (A) Rare visceral arterial branch injury (splenic artery branch) identified during angiographic evaluation. (B) Superior gluteal artery injury. (C) Obturator artery injury. (D) Internal pudendal artery injury.
